# Mitochondrial DNA 10158T>C mutation in a patient with mitochondrial encephalomyopathy with lactic acidosis, and stroke-like episodes syndrome: A case-report and literature review (CARE-complaint)

**DOI:** 10.1097/MD.0000000000020310

**Published:** 2020-06-12

**Authors:** Shuai Wang, Tao Song, Suping Wang

**Affiliations:** Department of neurological rehabilitation, The Third People's Hospital of Qingdao.

**Keywords:** M.10158T>C mutation, mitochondrial encephalomyopathy with lactic acidosis, and stroke-like episodes, mitochondrial disorder

## Abstract

**Rationale:**

Mitochondrial encephalomyopathy with lactic acidosis and stroke- like episodes (MELAS) syndrome is caused by mitochondrial respiratory chain dysfunction and oxidative phosphorylation disorder. It is a rare clinical metabolic disease involved with multiple systems.

**Patient concerns:**

A 22-year-old patient presented with limb convulsion accompanied by loss of consciousness, headache, partial blindness, blurred vision, and so on.

**Diagnoses:**

Brain magnetic resonance imaging showed a high-intensity area in bilateral occipital cortex, left parietal lobe and cerebellum on diffusion-weighted imaging. These focus did not distribute as vascular territory. The pathological examination of skeletal muscle revealed several succinate dehydrogenase reactive vessels with overreaction and increased content of lipid droplets in some muscle fibers. Genetic testing showed that the patient carried m.10158T>C mutation.

**Interventions:**

She was provided with traditional arginine hydrochloride therapy and orally medication of coenzyme Q (10 mg).

**Outcomes:**

Mitochondrial DNA of blood and hair follicle of patient carried m.10158T>C mutation

**Lessons:**

For the suspected patients of MELAS syndrome, if the hot-spot mutation test is negative, more detection sites should be selected.

## 1 Introduction

Mitochondrial encephalomyopathy with lactic acidosis and stroke- like episodes (MELAS) syndrome, a mitochondrial disease, is caused by mitochondrial respiratory chain dysfunction and oxidative phosphorylation disorder. It is a rare clinical metabolic disease involved with multiple systems,^[1,2]^ the clinical features of which includes mitochondrial myopathy, hyperlactacidemia and stroke-like episodes. Most infected patients are matrilineal-inherited cases and minor are sporadic cases. MELAS syndrome is firstly reported by Pavlakis in 1984.^[3]^ About 80% MELAS cases were cased by A>G mutation (A3243G) in 3243 locus of mitochondrial DNA (mtDNA).^[4,5]^ This mutation brings about new restriction site of ApaI, results in tRNA structure changes of leucine gene, affects protein synthesis and energy production in mitochondria, and eventually causes the occurrence of diseases. In recent years, more and more mtDNA mutations related to MELAS syndrome were detected, such as A3252G, T3271C, T3291C and G3959A.^[6]^ m.10158T>C is located in the loop domain of subunit (MT-ND3) of mitochondria complex I. It is rarely reported about the relationship of m.10158T>C mutation with MELAS syndrome. In this present report, we reported the clinical characteristics, imaging features, laboratory results, skeletal muscle pathological features and gene monitoring results of a MELAS case related with m.10158T>C mutation.

## 2 Materials and methods

### 2.1 Subject

The study was approved by the ethics committee of Third People's Hospital of Qingdao. The case is a female, 22-year-old teacher with normal development state, full-term normal delivery, normal pregnancy and suckling periods. Her parent is still living and no similar history of mitochondria and genetic diseases was recorded. The case was hospitalized when she was 19 years old due to physical convulsions with consciousness loss. Laboratory results were: pH 7.07 (normal value: 7.35–7.45), lactic acid 6.5mmol/L(normal value: 0.5–1.6 mmol/L), lactic dehydrogenase 342 IU/L (normal value: 120–250 IU/L), creatine kinase 1645 IU/L (normal value: 22–269 U/L). Punctiform long T2 and T1 signals at the right cerebellar hemisphere and right occipital lobe, unclear boundary, FLAIR and diffusion-weighted imaging (DWI) images with hyperintensity were observed in brain magnetic resonance imaging (MRI). She was provided with anti-epileptic and antiviral therapies and the symptoms were improved. She was hospitalized when 21 years old because of partial side headache with right-side hemianopsia and blurred vision. Laboratory results were: FLAIR and DWI images with hyperintensity, ADC images with hypointensity and normal CTA of head and neck, and brain MRV. After 10-d treatments, leaf patchy long T1 and T2 abnormal signals at left-side pillow top was improved, and then the case was supplied with therapy for improving circulation and best support care until out of hospital.

**Figure 1:**
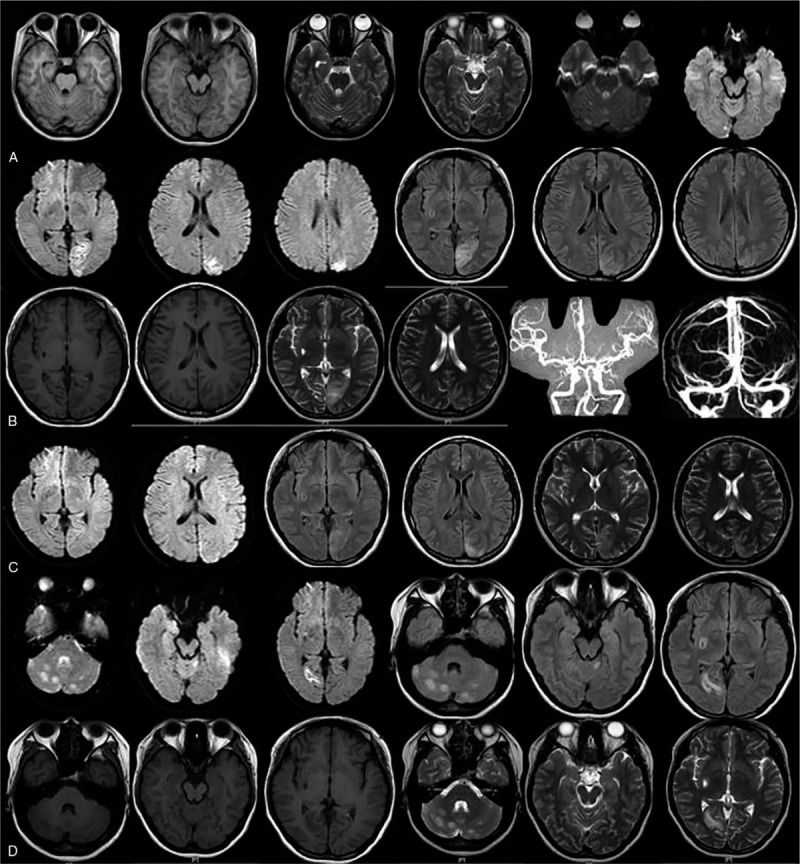
Image data of brain. A: The first hospitalization at 19 years old. Brain MRI showed punctiform long T2 and T1 signals at right cerebellar hemisphere and right occipital lobe, unclear boundary and DWI images with hyperintensity. B: At 21 years old. Brain MRI showed gyrus-shape long T1 and T2 abnormal signals at left-side pillow top and FLAIR and DWI images with hyperintensity. Brain MRV and CTA in head and neck showed no obvious abnormity. C: Reexamination after 10-day treatment. Brain MRI showed that gyrus shape long T1 and T2 signals at left occipital parietal lobe was improved. D: Recurrence at 22 years old. Brain MRI showed schistose and patchy signals at cerebellar hemispheres, vermis cerebelli, and right occipital area. MRI = magnetic resonance imaging.

The case was hospitalized again at 22 years old because of physical convulsions with consciousness loss. Laboratory results were: pH 7.01, lactic acid 9.9 mmol/L. Cerebrospional fluid cytology detection showed normal results. Brain MRI showed schistose and patchy high-signal shadow in DWI images of cerebellar hemispheres, vermis cerebelli, and right occipital area, slightly lowered signal of ADC image (Fig. 1). Electromyography revealed multiple peripheral neuropathy mainly caused by sensory axonal damage, injury of left peroneal nerve at the ankle, and myogenic damage at left biceps. Electroencephalogram (EEG) exhibited moderately abnormal state: epileptiform pattern, extraordinary wave, and occasional paroxysm of atypical spike and slow complex waves at left parietal, occipital and temporal regions. She is provided with traditional arginine hydrochloride therapy and orally medication of coenzyme *Q* (10 mg).

## 3 Methods

### 3.1 Skeletal muscle pathology detection

Biopsies of right biceps were obtained after the patient and her family member signed the informed consent. Frozen sections were then treated by hematoxylin and eosin staining, modified Gomori trichrome (MGT) staining, succinic dehydrogenase (SDH) staining, cytochrome coxidase (COX) staining, periodic acid Schiff reaction, adenosine triphosphatase staining and MHC-1 immunohistochemical staining, respectively.

### 3.2 mtDNA mutation analysis

Blood and hair from patient were sent to Bestnovo Medical Technology Co., Ltd (Beijing) and mtDNA mutation was analyzed. mtDNA was collected from whole blood and hair, and then was quantified. It was amplified via agarose gel eletrophoresis. The terminals of amplified fragments were treated by for construction of sequencing libraries. The DNA fragments were detected by Agilent2100 Bioanalyzer. The qualified samples were sequenced at Illumina NovaSeq platform. MAQ and BWA softwares were used to analyze the data.

## 4 Results

### 4.1 Clinical diagnosis

The patient was diagnosed as adult-onset MELAS syndrome according clinical features, imaging traits, laboratory results, skeletal muscle pathological characteristics and genetic detection.

### 4.2 Skeletal muscle pathology detection

According to HE staining, we found differed size and weight of muscle fiber and polygonal fibril. MGT staining showed more engrained regions under myolemma of muscle fibres. No tradtional ragged-blue fibers was observed in SDH staining, while three strongly succinate dehydrogenase reactive vessels (SSVs) and more engrained regions under myolemma of muscle fibres were observed. ORO staining suggested slightly increased content of fibrolipid droplets. COX staining indicated that all muscle fibres showed enzyme activity. Periodic acid schiff results suggested no obvious increase in glycogen content within muscle fibres, while it showed MHC-1 staining positive of multiple muscle fibres.

### 4.3 mtDNA mutation analysis

**Figure 2:**
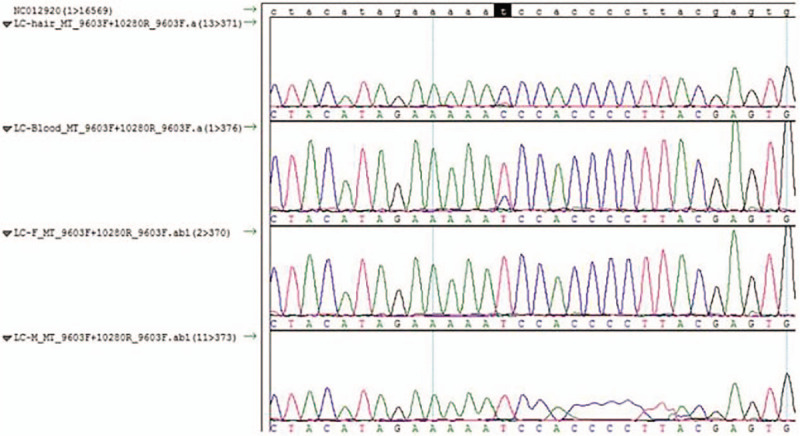
Sanger sequence diagram. Mitochondrial DNA of the patient, detected in serum and hair follicle, carried m.10158T>T/C mutation site. DNA = deoxyribose nucleic acid.

After detailed screening of mitochondrial genome, we found that mtDNA of blood and hair follicle carried m.10158T>C mutation (Fig. 2). There were no obvious abnormity in the mitochondrial genome of the parent of patient.

## 5 Discussion

Mitochondrial diseases is caused by the defects in mDNAs or nDNAs that encoded respiratory chain complex, more than 30% of which is caused by defect of respiratory chain complex I (NADH-ubiquinone oxidoreductase). Respiratory chain complex I is the biggest and most complicated calcium-calmodulin complex in the respiratory chain, including 7 nuclear subunits (MT-ND1–6 and MT-ND4L) encoded by mtDNA and 38 nuclear subunits encoded by nDNA.^[6]^ The defects in respiratory chain I could bring about multiple clinical diseases and MELAS syndrome is the most common 1.^[7]^ It is usually misdiagnosed because of complex and diverse clinical features without specificity and highly heterogeneous genotype. Thus, early diagnosis and timely treatment contribute to delaying disease progression and improving the prognosis. According to the clinical phenotype, muscle pathology examination and gene detection results of the patient and previously published articles, the features of this disease were summarized to deepen the understanding of MELAS syndrome and guide clinical work.

### 5.1 Clinical features

MELAS syndrome occurs in cases at any age, while it usually occurs before 40 years old.^[8]^ It commonly occurs in children and adolescent and sometimes occurs in old people. Its clinical features are as follows: a. Neurological involvement. Stroke-like episodes is the typical characteristic of MELAS syndrome. It shows different clinical symptoms based on different injured parts. The common clinical symptoms include hemiplegia, aphasia, hemianopsia, hemidysesthesia and disturbance of consciousness. In addition, epileptiform seizure and frequent headache are also common clinical symptoms of neurological involvement.^[9]^ The patients with MELAS syndrome also shows chronic progressive hearing impairment, which shows dropped reaction to high-frequency acoustic stimulation.^[10]^ Other neurological symptoms include peripheral neuropathy, cognitive decline, myoclonus, complication of eyes, and psychogeny.^[11]^ b. Muscle involvement. It mainly manifests exercise intoleranee (incidence rate: 73%-100%) and myasthenia (incidence rate: 42%-89%).^[11]^ c. Endocrine system involvement. The symptoms mainly include diabetes mellitus, short stature, and stunting due to growth hormone deficiency. Other symptoms include hypothyroidism, parathyroidism and hypogonadism.^[12]^ d. angiocarpy, digestive tract, urinary system, respiratory tract, and skin system involvement. The symptoms cover chronic heart failure, intestinal pseudo-obstruction, glomerulonephritis, and leucoderma.^[13]^ In the present case report, the patient exhibited epileptiform seizure, disturbance of consciousness, partial side headache, right-side hemianopsia and blurred vision. These symptoms repeatedly attacked and conformed to clinical manifestations of MELAS syndrome.

### 5.2 Laboratory assay and electro-physiological examination

Hyperlactacidemia is the marked feature of MELAS syndrome. The patient of MELAS syndrome always shows obvious rise of lactic acid and pyruvic acid contents in serum and cerebrospinal fluid. Mitochondrial dysfunction brings about incomplete oxidization of glucose, strengthened anaerobic glycolysis, pyruvate accumulation and lactic acid production. Additionally, blood lactic acid and pyruvate exercise test also show certain diagnostic value. The ratio of lactic acid and pyruvate, and blood level of lactic acid usually rise after exercises.^[14]^ Moreover, some MELAS patients exhibit increased serum level of creatine jubase and lactic dehydrogenase, and lowered activity of complex enzyme in mitochondrial respiratory chain. Serum lactic acid level was always high in the reported patient and serum creatine jubase and lactic dehydrogenase are slightly high during repeated course. Cerebrospional fluid cytology results were normal, while lactic acid content obviously increased in cerebrospinal fluid. All the results accord with MELAS syndrome.

Electro-physiological examination has no specificity in diagnosis of MELAS syndrome. Electromyography suggest that most patients have myogenic damages, minority have neurogenic damage or both damages, and some have normal results. EEG shows broad anomalous wave, localized ratchet complex waves and diffuse slow waves on both sides. It will be much more meaningful if EEG could reveal interictal epileptic discharge origin from the posterior cortex of the brain and the patients show photosensitive seizures. In this report, the patient was diagnosed with multiple peripheral neuropathy mainly related to sensory axonal injury, left common peroneal nerve injury at ankle, and myogenic injury of the left biceps brachii. EEG showed moderate abnormity, epileptiform pattern, occasional paroxysm of atypical spike and slow wave complex at left parietal, occipital and temporal regions.

### 5.3 Imaging characteristics

MELAS syndrome has particular imaging characteristics, which may provide important reference value for the diagnosis of MELAS. The focus often attacks and mainly involves posterior of both-side hemispheres (temporal, parietal, occipital lobes), epencephalon, and basal ganglia region usually limited to cortical and subcortical white matter, which show gyriform abnormalities signal shadow.^[15]^ MRI detection at acute stage show broad long T1 and long T2 signals, FLAIR and DWI with hyperintensity, inconformity of focus distribution and cerebrovascular supply area (no evidence of cerebral atherosclerosis or stenosis and no intensify or string intensify in enhanced MRI). MRS examination suggest lactate peak and reduced level of N-acetylaspartate. In our report, the patient repeatedly presented long L1 and long L2 abnormal signal at bilateral occipital lobe, left parietal lobe and epencephalon detected by brain MRI in progress, as well as the high-intensity FLAIR and DWI images. With the progression of disease, original focuses were decreased and some were vanished. No obvious abnormity was observed in the head and neck CTA, and brain MRV, while the focuses were not distributed according to vascular regions. All these imaging characteristics met the criterion of MELAS syndrome.

### 5.4 Muscle pathological examination

Pathological examination of muscle tissues is valuable for diagnosis of MELAS syndrome. The typical characteristics of MELAS patients in muscle pathological detection were a large number of ragged red fiber (RRF) by MGT staining, engrained ragged-blue fibers and SSVs by SDH staining, increased content of fibrolipid droplets by ORO staining, activity deficiency of some enzymes by COX staining, abnormal accumulation of mitochondria under myolemma or between the myofibrils, disorganized mitochondrial cristae, and crystalloid inclusion body. The reported patient showed 3 SSVs and engrained regions under myofiber myolemma by SDH staining, slightly increase of fibrolipid droplets by ORO staining, no RRF by MGT staining, and no activity deficiency of enzymes. The previously published studies indicated that no RRF in muscle biopsy did not mean the absence of MELAS syndrome and about 10% of MELAS patients showed no RRF in muscle biopsy.^[16]^ Due to the heterogeneity of mtDNA mutation, involvement degrees of brain and muscle may be varied. In addition, RRF is not unique in MELAS patients and it may be present in other diseases, such as myotonic muscular dystrophy, inclusion body myositis and healthy old people above than 50 years old. The presence of SSVs is regarded as much more specific biomarker than RRF in the diagnosis of MELAS, which fully reflects the pathological changes in small vessel of MELAS patients. Previous studies reported that MELAS patients caused by ND gene mutation were always deficient of RRFs, while the frequency of SSVs were relatively higher.^[17]^ In our report, the patient was confirmed with ND3 gene mutation, who showed same characteristic as the mentioned study. In addition, 1 study found that the muscle biopsy might be normal of certain MELAS patients caused by ND gene mutation.^[18]^

### 5.5 Genetic detection

MELAS syndrome is 1 of mitochondrial diseases with high heterogeneity in genotype. Genetic detection serves as an accurate diagnosis method for MELAS syndrome. About 80% MELAS cases were cased by A>G mutation (A3243G) in 3243 locus of mtDNA of leucine gene, while the relationship of m.10158T>C mutation and MELAS is rarely reported, which is not reported among Chinese population. The reported patient was confirmed to carry m.10158T>C mutation in mitochondrial genome. m.10158T>C in this report was located at loop domain of subunits of mitochondrial complex I (MT-ND3). This mutation could decrease the activity of complex I to 8% to 23% of that in control group. The frequency of this mutation is varied in different samples (muscle, blood, hair and fibroblast), the mutation of which is 73% to 100% in muscle and 0% to 48% in blood.^[18]^ The mutation is reported in MITOMAP database to relate with Leigh syndrome/MELAS, while it is not reported in China. In addition, the published study reported m.10158T>C showed much higher sporadic incidence than other mutations in mtDNA.^[8]^ In the present report, the parent of patient showed no abnormity in mitochondrial genome, thus, the patient was sporadic case.

### 5.6 Treatment and prognosis

Until now, there is no radically effective treatments for MELAS syndrome. The treatments include expectant treatment, gene therapy, sports rehabilitation training, and dietotherapy (ketogenic diet at acute stage). of note, valproates antiepileptic drugs should be avoid to control the epileptic seizure, since these drugs could damage proton pump of cytochrome oxidase, damage the ultrastructure of mitochondria, disturb oxidative phosphorylation process, and even result in hepatic failure of patients because of hepatotoxic side effect. Melbine DMBG also should not be used to avoid aggravating the symptoms of lactic acidosis when choosing hypoglycemic agents. These common drugs increasing mitochondrial function should be recommended, such as Q10, vitamin B complex, vitamin C complex, vitamin E complex, L-arginine, idebenone and modified cocktail therapy.^[19,20]^ Now, gene therapy is still in the research phase and it may be a more promising effective treatment direction in the future.

Above all, the reported patient exhibited physical convulsions with consciousness loss, headache, hemianopsia, and caligo, which is easily misdiagnosed as encephalitis at early phase. The physicians should carefully question the disease history of patients, conduct imaging and laboratory examinations, identify the course early according to the genetic detection results. Genetic detection technology could improve early diagnosis rate of MELAS syndrome, however, more mutation locus should be detected in cases highly suspicious of MELAS even if the hot mutation test is negative, since of high heterogeneity of MELAS syndrome. m.10158T>C mutation should be paid much more attention to improve the diagnosis rate.

## Acknowledgments

We are appreciated with the patient in the study.

## Author contributions

**Conceptualization:** Suping Wang.

**Data curation:** Shuai Wang.

**Formal analysis:** Shuai Wang, Suping Wang.

**Investigation:** Shuai Wang.

**Methodology:** Shuai Wang, Tao Song, Suping Wang.

**Software:** Tao Song.

**Supervision:** Suping Wang.

**Visualization:** Shuai Wang.

**Writing – original draft:** Shuai Wang.

**Writing – review and editing:** Tao Song, Suping Wang.
